# Infochemical use and dietary specialization in parasitoids: a meta‐analysis

**DOI:** 10.1002/ece3.2888

**Published:** 2017-05-25

**Authors:** Louise van Oudenhove, Ludovic Mailleret, Xavier Fauvergue

**Affiliations:** ^1^ Université Côte d'AzurINRACNRSISASophia AntipolisFrance; ^2^ Université Côte d'AzurINRIAINRACNRSUPMC Univ. Paris 06Sophia AntipolisFrance

**Keywords:** dietary breadth, foraging behavior, herbivore‐induced plant volatiles, information use, parasitoid, tritrophic interactions

## Abstract

Many parasitoid species use olfactory cues to locate their hosts. In tritrophic systems, parasitoids of herbivores can exploit the chemical blends emitted by plants in reaction to herbivore‐induced damage, known as herbivore‐induced plant volatiles (HIPVs). In this study, we explored the specificity and innateness of parasitoid responses to HIPVs using a meta‐analysis of data from the literature. Based on the concept of dietary specialization and infochemical use, we hypothesized that (i) specialist parasitoids (i.e., with narrow host ranges) should be attracted to specific HIPV signals, whereas generalist parasitoids (i.e., with broad host ranges) should be attracted to more generic HIPV signals and (ii) specialist parasitoids should innately respond to HIPVs, whereas generalist parasitoids should have to learn to associate HIPVs with host presence. We characterized the responses of 66 parasitoid species based on published studies of parasitoid behavior. Our meta‐analysis showed that (i) as predicted, specialist parasitoids were attracted to more specific signals than were generalist parasitoids but, (ii) contrary to expectations, response innateness depended on a parasitoid's target host life stage rather than on its degree of host specialization: parasitoids of larvae were more likely to show an innate response to HIPVs than were parasitoids of adults. This result changes our understanding of dietary specialization and highlights the need for further theoretical research that will help clarify infochemical use by parasitoids.

## 1 Introduction

**Figure 1:**
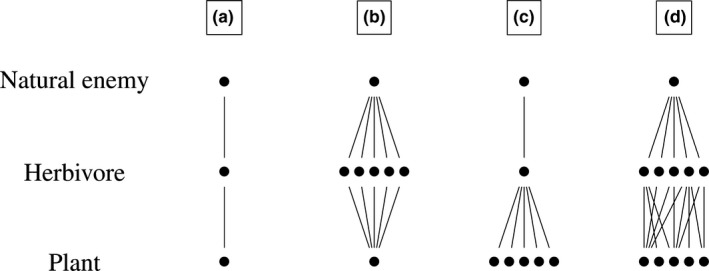
Different dietary specialization categories in a tritrophic (plant–herbivore–natural enemy) system: (a) specialist at both the herbivore and plant levels; (b) generalist at the herbivore level and specialist at the plant level; (c) specialist at the herbivore level and generalist at the plant level; (d) generalist at both the herbivore and plant levels. From Vet and Dicke (1992)

In plant–herbivore–carnivore tritrophic systems, different chemical cues might be used by carnivores to locate their herbivore prey/hosts. These infochemicals can be produced by the plant (i.e., via constitutive or herbivore‐induced expression) or by the herbivores themselves. Vet and Dicke (1992) hypothesized that the use of infochemicals by carnivores should evolve in response to dietary specialization. They placed carnivores in discrete classes based on their degree of prey/host specialization (Figure 1) and then generated the following predictions: (i) specialists at the plant level (A–B) should be innately attracted to infochemicals produced by the plant; (ii) specialists at the herbivore level and generalists at the plant level (C) might not display innate attraction but rather learn to associate infochemicals with their target prey/hosts; (iii) more specialized species should rely more on infochemicals to locate their prey/hosts; and (iv) extreme generalists (D) should not use infochemicals at all. Steidle and van Loon (2003) tested Vet and Dicke (1992)'s predictions using data from the literature on infochemical use by carnivorous arthropods. Their results supported hypotheses (ii) and (iii): associative learning was more common in generalists than in specialists and specialists used more specific cues, while generalists used more general cues. In contrast, Steidle and van Loon (2003) found no evidence to support hypotheses (i) and (iv). In their dataset, 29 extreme generalists used infochemicals, and 80% of these species were innately attracted to chemical cues from either the plant or the host(s). They suggested that the use of infochemicals was more efficient than random searching, and that even extreme generalists needed an innate set of reliable cues (Allison & Hare, 2009; Steidle & van Loon, 2003). In the present study, we aimed to explore the relationship between dietary specialization and infochemical use by focusing on parasitoids and the chemical blends that plants produce as a result of herbivore damage.

When certain plants are damaged by herbivorous arthropods, as a result of feeding or egg deposition for example, they release chemical blends that attract the herbivores’ natural enemies, including predators or parasitoids. Among the range of existing infochemicals, Vet and Dicke (1992) considered such herbivore‐induced plant volatiles (HIPVs) to provide the most specific cues. Indeed, HIPVs can be highly specific, signaling the presence of a particular herbivore species (DeMoraes, Lewis, Pare, Alborn, & Tumlinson, 1998) or even its specific life stage, in the case of phytophagous insects (Takabayashi, Takahashi, Dicke, & Posthumus, 1995). However, some HIPVs do not appear to be herbivore specific and instead trigger a generic response by predators or parasitoids \ (Hare, 2011; Kessler & Baldwin, 2001; Turlings, Mccall, Alborn, & Tumlinson, 1993). Therefore, the responses of natural enemies can present different degrees of specificity depending on signal specificity.

Since their discovery in the 1990s (Dicke, Sabelis, Takabayashi, Bruin, & Posthumus, 1990; Turlings, Tumlinson, & Lewis, 1990), HIPVs have been observed in a wide range of tritrophic systems (Dicke, 1999; Hilker & Meiners, 2002; Mumm & Dicke, 2010; Reddy, 2012). From insectivorous birds (Amo, Jansen, van Dam, Dicke, & Visser, 2013) to entomopathogenic nematodes (Van Tol et al., 2001), a broad variety of natural enemies use HIPVs to locate their prey/hosts. In particular, many predaceous and parasitoid insect species are attracted to HIPVs. For parasitoids, which depend directly on phytophagous hosts to reproduce, HIPVs are an effective way of bypassing the reliability–detectability problem as (i) the chemical signal is emitted by the plant and is thus not subject to selection for low detectability, as host cues might be and (ii) the signal can be highly specific, betraying the presence of a specific herbivore host (Vet & Dicke, 1992; Vet, Wackers, & Dicke, 1991). Parasitoids differ in their behavioral responses to HIPVs: in some species, attraction is innate (DeMoraes et al., 1998; Yan, Yan, & Wang, 2005), while in others, individuals must first learn to associate HIPVs with a given host–plant complex (Grasswitz, 1998; McCall, Turlings, Lewis, & Tumlinson, 1993). Moreover, some species only respond to HIPVs induced by their particular hosts (DeMoraes et al., 1998; Du, Poppy, & Powell, 1996), while others are attracted to HIPVs released by plants attacked by nonhost herbivores or by artificially damaged plants (Turlings et al., 1993; Yan et al., 2005). Additionally, some species only respond to HIPVs associated with particular host life stages (Colazza et al., 2004; Takabayashi et al., 1995), while others are attracted to HIPVs associated with different host life stages, including stages they cannot parasitize (Fatouros et al., 2012; Moraes, Laumann, Sujii, Pires, & Borges, 2005).

In this study, we revisited the concept of dietary specialization and infochemical use (Vet & Dicke, 1992) using parasitoids and HIPVs as a study system. Our aim was to understand how parasitoids respond to HIPVs and the different mechanisms underlying their responses. To characterize parasitoid behavior, we examined two key traits: response specificity and response innateness. We tested the association between these two traits and certain parasitoid life‐history traits. In particular, we looked at parasitoid host specialization, host dietary breadth, target host life stage, mean lifespan, and egg‐laying pattern. We tested five predictions. First, specialist parasitoids (Figure 1a,c) should respond to highly specific HIPVs, while generalist parasitoids (Figure 1b,d) should respond to more generic HIPVs. Second, parasitoids whose host(s) are dietary specialists (Figure 1a,b) should show an innate response, while parasitoids whose host(s) are dietary generalists (Figure 1c,d) should show a learned response. Third, parasitoids that attack nonfeeding host life stages (e.g., eggs) should show a learned response. Fourth, short‐lived parasitoids should show an innate response because they might not have the time to develop a learned response. Fifth, gregarious parasitoids should show an innate response as they might lay most of their eggs on a single host.

## 2 Materials and Methods

We searched for relevant references in the Web of Science, in the Science Citation Index Expanded^TM^ database. All articles published before 15 September 2016 and matching the following queries were considered: (response or behavior) and (parasitoid or parasitic) and (herbivore and induced and plant and volatiles). We found 393 articles, 49% of which dealt specifically with parasitoid behavior. Some cross‐references were added when pertinent. Among these publications, we focused on those concerning choice experiments involving mated females and in which non‐GM plants were damaged under controlled conditions. Ultimately, we were able to describe the behavior of 73 species of primary parasitoids.

We characterized the specificity and innateness of parasitoid responses to HIPVs in a binary fashion. Specificity conveyed the degree to which a parasitoid's response was specific versus generic. Specificity could be described for 37 parasitoid species. A response was defined as specific when a parasitoid was attracted to HIPVs released by a plant attacked by a host species (*n *= 23). A response was defined as generic when a parasitoid was attracted to HIPVs released by a plant damaged by a nonhost or by an artificially damaged plant (*n *= 14). Innateness conveyed the degree to which a response was innate versus learned. Innateness could be described for 63 parasitoid species. A response was defined as innate when a parasitoid was attracted to HIPVs without having had previous oviposition experience with a plant–host complex (*n *= 53). A response was defined as learned when a parasitoid needed such experience before being able to respond to related HIPVs (*n *= 10). For seven species, neither specificity nor innateness could be described: the related studies tested interactions between experienced individuals and plants damaged by hosts. The species were consequently excluded from the meta‐analysis.

The focal life‐history traits were determined for the other 66 parasitoid species (Appendix S1): (i) degree of host specialization: generalist (attacks hosts of different taxonomic families (Stireman & Singer, 2003); *n *= 29), oligophage (attacks hosts of single family but multiple subfamilies; *n *= 9), or specialist (attacks hosts of one subfamily; *n *= 28); (ii) host dietary breadth: broad (host[s] attack different taxonomic families of plants; *n *= 48) or narrow (host[s] attack plants of one family; *n *= 18); (iii) target host life stage: egg (*n *= 17), larva (*n *= 41), or adult (*n *= 8) (parasitoids of pupae were grouped with parasitoids of larvae); (iv) lifespan (continuous variable with 14 missing values); and (v) egg‐laying pattern: solitary (*n *= 52) or gregarious (*n *= 14).

The parasitoid species we examined (Appendix S1) belonged to two different orders: Diptera (*n *= 3) and Hymenoptera (*n *= 63). Their degrees of relatedness were thus highly variable. To avoid bias in our results due to phylogenetic autocorrelation, we accounted for phylogenetic relationships in our analyses. To this end, a phylogenetic tree of the study species was built using the phylogenetic trees found in the literature for the following taxonomic groups: the order Hymenoptera (Davis, Baldauf, & Mayhew, 2010); the families Ichneumonidae and Braconidae (Quicke, 2015); the family Eulophidae (Burks, Heraty, Gebiola, & Hansson, 2011); the subfamily Aphidiinae (Sanchis, Latorre, González‐Candelas, & Michelena, 2000); the subfamily Opiinae (Wharton, Yoder, Gillespie, Patton, & Honeycutt, 2006); the subfamily Microgastrinae (Mardulyn & Whitfield, 1999); and the subfamily Exoristinae (Tachi & Shima, 2010). It must be noted that the resulting tree (Appendix S2) does not have interpretable branch lengths as the criteria used in the different source publications were not equivalent.

To remove phylogenetic autocorrelation (Appendix S2), autoregressive models were used (Cheverud, Dow, & Leutenegger, 1985). First, Abouheif's matrix of phylogenetic proximities was built (Pavoine, Ollier, Pontier, & Chessel, 2008): this matrix provides a measure of phylogenetic relatedness between species pairs that does not account for branch length. Then, a lag vector was defined for each response trait (i.e., specificity and innateness) using the phylogenetic proximities matrix. The lag vector represented the variation in the response that was explained by phylogeny (Appendix S2). Each of the two response traits was then analyzed independently by fitting a generalized linear model (GLM) with a logit link function and binomial error. The explanatory variables were the response‐specific lag vector and the focal life‐history traits. Nonsignificant life‐history traits were removed in a stepwise fashion from the model based on chi‐square tests of residual deviances. Analyses were performed using R v. 3.1.1 (R Development Core Team, 2010), and the package adephylo 1.1‐6 (Jombart, Balloux, & Dray, 2010).

## 3 Results

### 3.1 Response specificity

Among the study species, all those with sufficient oviposition experience responded to HIPVs induced by damage caused by their hosts. However, some species were also attracted to HIPVs produced following nonhost damage, thus displaying a generic response.

**Figure 2:**
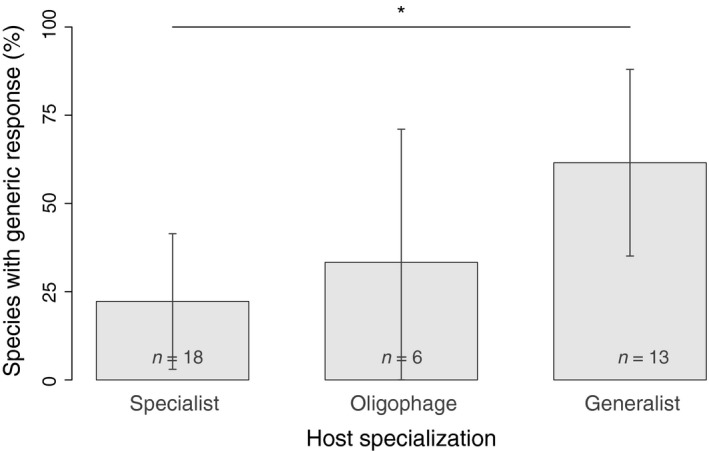
Percentage of species with generic responses (i.e., parasitoids that were attracted by HIPVs induced by nonhost or artificial damage) according to the degree of host specialization. The bars represent observed percentages with 95% confidence intervals (*n* is specified for each category). The star represents significant difference according to the final GLM (with correction for phylogenetic autocorrelation)

As expected, response specificity was strongly linked to the degree of parasitoid host specialization (Table 1). Specialist parasitoids tended to respond to specific signals produced by plants attacked by their host(s). Indeed, about 78% of specialist parasitoids failed to respond to signals emitted by plants damaged by at least one nonhost herbivore or artificially damaged (Figure 2). Generalists showed very different behavior (*t *=* *2.31, *p *=* *.02). They tended to respond to generic signals: most (about 62%) were also attracted by HIPVs released by plants attacked by nonhosts, or by artificially damaged plants (Figure 2). The behavior of oligophages did not differ from that of specialists (*t *= 0.59, *p *= .56) or generalists (*t *= 1.29, *p *= .20). They showed an intermediate level of response specificity: about 67% of oligophagous species responded only to damage caused by their hosts, while 33% also responded to generic signals (Figure 2). Host dietary breadth was not correlated with response specificity (Table 1, Appendix S3).

**Table 1:** Relationship between life‐history traits and HIPV response specificity and innateness. Response specificity and innateness were dependent variables in separate GLMs in which the life‐history traits were the explanatory variables (with correction for phylogenetic autocorrelation). Variable effects were tested with χ^2^ tests on deviance reduction

Life‐history traits	Response specificity			Response innateness		
	Deviance reduction	*df*	*p‐*value	Deviance reduction	*df*	*p*‐value
Parasitoid host specialization	6.24	2	.04	1.67	2	.43
Host dietary breadth	0.94	1	.33	0.01	1	.91
Target host stage	5.24	2	.07	6.63	2	.04
Lifespan	3.56	1	.06	1.10	1	.30
Egg‐laying pattern	3.57	1	.06	3.06	1	.08

The other life‐history traits were not correlated with response specificity (Table 1). The percentage of parasitoid species attracted to generic HIPVs were similar regardless of lifespan or egg‐laying pattern (Appendix S3). With regard to target host life stage, no parasitoid of adults presented a generic response to HIPVs (Appendix S3). However, we only had response specificities for three parasitoids of adults. Indeed, we had a probability of 0.4 of observing this pattern if the prevalence of species attracted by generic signals was identical among parasitoids of adults and of larvae, which means this observation might be due to the small sample size.

### 3.2 Response innateness

All the study species responded to HIPVs associated with damage caused by their hosts. In some species, this response was innate: females were attracted to HIPVs without having had any previous oviposition experience with a given plant–host complex. In other species, females had to learn to associate HIPVs with plant–host complexes via oviposition experience. Response innateness was not associated with response specificity (Fisher's exact test: *p *=* *.63).

**Figure 3:**
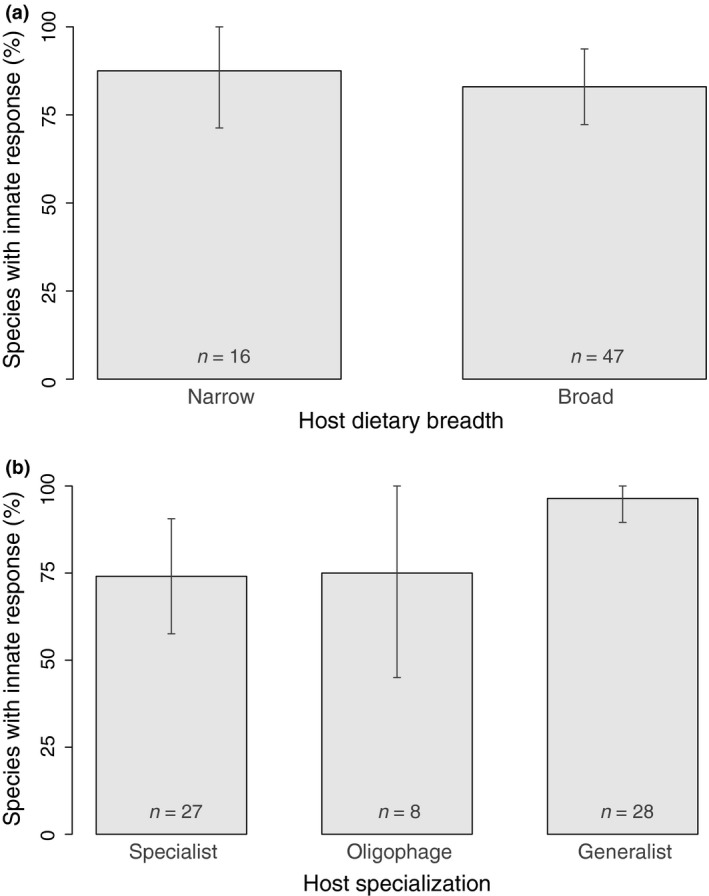
Percentage of species displaying innate responses according to (a) the range of host dietary breadth; (b) host specialization. The bars represent the observed percentages with 95% confidence intervals (*n* is specified for each category)

Contrary to what was hypothesized, response innateness was linked neither to parasitoid host specialization nor to host dietary breadth (Table 1). Host dietary breadth was not correlated with response innateness (Table 1, Figure 3a), nor did generalists, oligophages, or specialists differ in response innateness (Table 1, Figure 3b).

**Figure 4:**
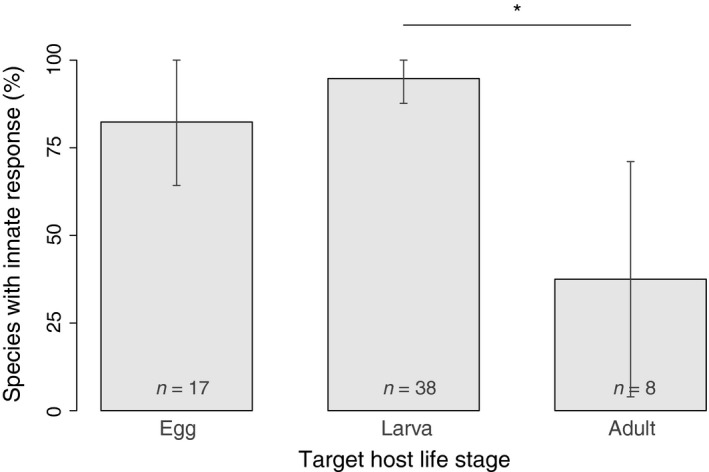
Percentage of species displaying innate responses according to the target host life stage. The bars represent observed percentages with 95% confidence intervals (*n* is specified for each category). The star represents significant difference according to the final GLM (with correction for phylogenetic autocorrelation)

In contrast, response innateness was associated with target host life stage (Table 1). About 95% of the parasitoids of larvae/pupae responded innately to HIPVs (Figure 4). Fewer parasitoids of adults displayed an innate response (*t *= 2.28, *p *= .03): only 37.5% responded to HIPVs without having had previous oviposition experience with a given plant–host complex. The remaining 62.5% needed to learn the association. Although more than 80% of the parasitoids of eggs responded innately to HIPVs (Figure 4), this number was not significantly different than those for parasitoids of larvae (*t *= 1.36, *p *= .18) and of adults (*t *= 1.53, *p *= .13), once phylogenetic autocorrelation and sample size were taken into account.

**Figure 5:**
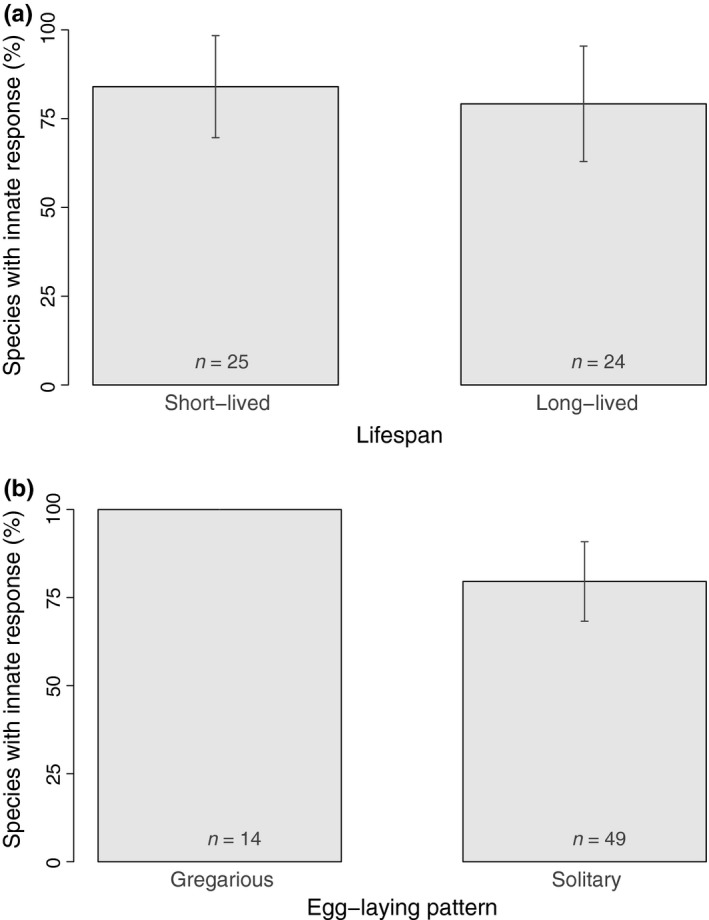
Percentage of species displaying innate responses according to (a) lifespan (transformed into a two‐category variable for illustrative purposes; cut at the median); (b) egg‐laying pattern. The bars represent the observed percentages with 95% confidence intervals (*n* is specified for each category)

There was no support for our hypothesis that short‐lived parasitoids would be more likely to respond innately than long‐lived parasitoids: response innateness was not associated with lifespan (Table 1, Figure 5a).

Egg‐laying patterns were not significantly correlated with response innateness (*p *= .08 in Table 1). However, in general, innate responses were more common among gregarious parasitoids than among solitary parasitoids (Figure 5b). Indeed, 100% of gregarious species versus 80% of solitary species displayed innate responses to HIPVs, and gregarious species represented 21% of the 66 study species. If response innateness was the same for gregarious and solitary species, the probability of observing such a pattern would be low (0.04), which supports the validity of the trend.

## 4 Discussion

Using data from the literature, we characterized how 66 parasitoid species responded to HIPVs and tested Vet and Dicke (1992)'s predictions. Our main conclusions are the following: (i) specialist parasitoids responded to highly specific HIPVs, while generalist parasitoids responded to more generic HIPVs; (ii) specialist parasitoids whose hosts have a narrow dietary breadth did not display greater response innateness than did parasitoids whose hosts have greater dietary breadth; (iii) response innateness was similar between parasitoids that attack nonfeeding host life stages (i.e., parasitoids of eggs) and other parasitoids; (iv) there was no correlation between parasitoid lifespan and either response specificity or innateness; and (v) gregarious parasitoids tend to show more of an innate response than do solitary parasitoids. We also observed an unexpected result: (vi) innate responses were less common in parasitoids of adults than in parasitoids of larvae.

Our results refute the main prediction of Vet and Dicke (1992)'s theory regarding infochemicals and dietary breadth: we found that about 85% of parasitoid species were innately attracted to HIPVs and that response innateness was not correlated with the range of plants consumed by their hosts. Moreover, innate responses were just as common in specialists as in generalists. This finding is consistent with Steidle and van Loon (2003)'s general conclusions about infochemicals (whether produced by the host or the plant): host dietary breadth is not a determinant of parasitoid response innateness.

Nevertheless, Vet and Dicke (1992)'s general prediction regarding the relationship between the degree of host specialization and the specificity of the information needed to forage successfully was supported by our results for parasitoids and HIPVs. Specialist parasitoids only responded to HIPVs released in response to damage by their herbivore host(s), while generalist parasitoids also responded to HIPVs released following damage by nonhost herbivores or artificial damage. This relationship between host specialization and response specificity seems to follow a gradient because response specificity was intermediate in oligophagous parasitoids: essentially, the more generalist the parasitoid, the less specific the response. This pattern is present at an even larger scale; it also describes the relationship between the diversity of carnivorous species and the range of infochemicals (Steidle & van Loon, 2003).

It has been hypothesized that parasitoids of nonfeeding life stages should not employ HIPVs because such host stages do not physically damage the plant. However, many studies have shown that plants do react to ovipositioning by herbivorous insects and release HIPVs that might be attractive to parasitoids of eggs (Hilker & Fatouros, 2015; Hilker & Meiners, 2002). Furthermore, some parasitoids of larvae are also attracted by these same blends (Fatouros et al., 2012). Indeed, our results show that parasitoids of eggs did not differ in their responses from parasitoids of feeding life stages. There was, nevertheless, a correlation between response innateness and target host stage, just not in the expected direction. Rather, we observed a difference between parasitoids of larvae/pupae and parasitoids of adults. The former were more likely to show an innate response than were the latter. This might be explained by host motility: as adults are far more motile, plant volatiles released following herbivore damage at time *t* might not necessarily reveal an adult's position at time *t *+ 1. Parasitoids of adults might be better off focusing on volatiles emitted directly by the host (e.g., sex pheromones).

Olfactory learning occurs at different stages of insect ontogeny (Gandolfi, Mattiacci, & Dorn, 2003). In parasitoids, which spend their preimaginal stages in/on their host, it is difficult to determine whether individuals are naive or have learned to recognize chemical cues (Allison & Hare, 2009). In this study, we considered a female to be naive if she had no prior oviposition experience on her host(s). However, the pre‐emergence stages seem to be critical in establishing a parasitoid's attraction to HIPVs. Indeed, Takemoto, Powell, Pickett, Kainoh, and Takabayashi (2012) demonstrated that the parasitoid *Aphidius ervi* needed preimaginal exposure to HIPVs induced by host aphids to be attracted to HIPVs post emergence. Moreover, response specificity might also be affected by preimaginal experience. For instance, when reared on Brussels sprouts *Brassica oleracea*, both the specialist *Diadegma semiclausum* and the generalist *D. fenestrale* were attracted by HIPVs induced by nonhosts but did not distinguish among nonspecific HIPVs released by other brassicaceous plant species (Gols et al., 2012). Here, we considered that a response was learned when oviposition experience shifted a response from *nonsignificant* to *attraction*. However, the definition of learning might need to be more specific than “behavioral change as a result of experience” as a “learned response can be forgotten […] as a consequence of another experience” (Vet & Lewis, 1995). Response forgettability has rarely been studied in the context of parasitoid attraction to HIPVs but might be an important aspect of foraging behavior plasticity, especially in long‐lived species. Indeed, associative learning might be more frequently employed in long‐lived parasitoids than in short‐lived parasitoids, as the latter exploit a small number of host patches during their live (Vet et al., 1991). We had therefore expected responses by short‐lived parasitoids to be innate, but response innateness was not tied to lifespan in our study species. However, we did observe an association between response innateness and egg‐laying pattern. As expected, gregarious parasitoids were more likely than solitary parasitoids to display innate response. Learning dynamics should vary between gregarious or quasi‐gregarious and solitary species because (i) gregarious parasitoids might not acquire sufficient oviposition experience during their lives and (ii) the value of host patches is higher for gregarious species, who may lay many eggs on/in a single individual host (Hoedjes et al., 2011).

The terms “specialist” and “generalist” are helpful when formulating ecological concepts, but debate over their definitions is far from resolved (Finlay‐Doney & Walter, 2012). Parasite success is the result of many different steps, from host location to host regulation (Vinson, 1976). Consequently, the degree of host specialization is not solely determined by host suitability; the direct and indirect interactions occurring between parasitoids and hosts in local communities must also be considered (Finlay‐Doney & Walter, 2012; Fox & Morrow, 1981). Specialization is defined in different ways in predaceous arthropods, with the definition involving either prey species number or diversity (Finlay‐Doney & Walter, 2012). In this study, we defined three categories (Vet & Dicke, 1992): generalists (attack hosts from more than one taxonomic family), oligophages (attack hosts from a single taxonomic family), and specialists (attack hosts from a single subfamily) (Stireman & Singer, 2003). We chose not to focus on host number, which is notoriously difficult to estimate and sensitive to the research effort that has been deployed for a given species. However, this categorization system remains somewhat arbitrary as species with many hosts in a given subfamily can be called specialists (e.g., *A. ervi*; Thompson, 1953), while species with a few hosts scattered across different families can be called generalists (e.g., *Telenomus podisi*; Thompson, 1958). Nevertheless, even using this rough classification scheme, we observed a link between parasitoid host specialization and response specificity, supporting the idea that specialization results from physiological and behavioral interactions between parasitoids and hosts in particular environments (Finlay‐Doney & Walter, 2012; Vinson, 1976).

Of the publications examined during this study, very few reported that parasitoids with prior oviposition experience failed to respond to HIPVs. This observation raises the following question: are our results strongly affected by the publication bias against negative results (Thornton & Lee, 2000)? Or does it mean that all parasitoid species can detect HIPVs? Buitenhuis, Vet, Boivin, and Brodeur (2005) reported that experienced mated females were not attracted to HIPVs in four hyperparasitoid species. The authors concluded that hyperparasitoids did not rely on chemical cues to locate their hosts, but this generalization appears to be false (Poelman et al., 2012). Similarly, Charleston et al. (2006) found that experienced *Diadromus collaris* did not distinguish between undamaged and caterpillar‐damaged cabbage plants. However, the experimental setup used might have influenced species response behavior. In particular, the time that nonresponding individuals were left in the experimental arena before being removed might have been crucial. Evidence for this concern comes from two other studies: naive *Exorista japonica* females were not attracted by host‐damaged corn plants when left for 2 min in a wind tunnel (Kainoh, Tanaka, & Nakamura, 1999) but they were when left for 5 min (Ichiki, Kainoh, Kugimiya, Takabayashi, & Nakamura, 2008). Likewise, naive *Trichogramma brassicae* females were not attracted by *Pieris brassicae*‐damaged Brussels sprouts when single individuals were left for 5 min in a Y‐olfactometer (Fatouros et al., 2005), but 10 individuals left for 30 min in the same device were significantly attracted by *Brassica nigra* damaged by *P. brassicae* (Fatouros et al., 2012).. Ambient conditions might also have a substantial influence (Pinto et al., 2007; Vuorinen, Nerg, Ibrahim, Reddy, & Holopainen, 2004). More importantly, many parasitoids show different responses depending on the specific plant–host complex (Ero & Clarke, 2012; Gols et al., 2012; Krugner, Johnson, Daane, & Morse, 2008; Whitman & Eller, 1990) or even plant variety (Raghava, Ravikumar, Hegde, & Kush, 2010; Tamiru et al., 2011). Therefore, study conclusions could change based on different experimental protocols, experimental devices, and host–plant complexes (see also the discrepancies for *Aphidius colemani*, (Grasswitz, 1998; Lo Pinto, Wajnberg, Colazza, Curty, & Fauvergue, 2004), and *Cotesia vestalis*, (Potting, Poppy, & Schuler, 1999; Shiojiri et al., 2000)). It is therefore difficult to definitively conclude that a species is not attracted by HIPVs.

Contrary to the predicted relationship between dietary specialization and infochemical use, response innateness was not associated with host specialization in parasitoids. Our results suggest almost all parasitoid species use HIPVs as a reliable cue of host presence. Interspecific differences in response innateness may depend instead on the cue's ability to signal available hosts. For instance, depending on host motility, parasitoids might need to use associative learning to confirm the signal's reliability. These differences in response innateness might also be tied to the value of a given host patch for a given parasitoid. For example, innate responses might be especially adaptive when host patches are clustered and/or parasitoids are gregarious.

## Conflict of Interest

None declared.
