# Supplementary material for: Infochemical use and dietary specialization in parasitoids: a meta‐analysis
Source: Ecol Evol. 2017 May 25;7(13):4804–11. doi: 10.1002/ece3.2888 (PMC5496531; doi:10.1002/ece3.2888)
Supplement: Supplementary file 1 [file ECE3-7-4804-s001.pdf]

Infochemical use and dietary specialization in parasitoids: a meta-analysis

Louise van Oudenhove [1], Ludovic Mailleret [1,2], Xavier Fauvergue [1]

(1) Université Côte d'Azur, INRA, CNRS, ISA, France.

(2) Université Côte d'Azur, INRIA, INRA, CNRS, UPMC Univ. Paris 06, France.

A Documented species

Table A.1: List of study species: parasitoids for which HIPV response specificity or response innateness was characterized. We only looked at choice experiments involving mated females tested with non-GM plants damaged under controlled conditions. The references are not exhaustive.

Species	Innateness	Specificity	Reference(s) for response behavior	Host specificity	Host dietary breadth	Reference for dietary breadth	Target host life stage	Lifespan	Reference for lifespan	Egg-laying pattern
**Hymenoptera: Aphelinidae**										
Aphelinus abdominalis (Dalman)	learned	.	(Molck et al., 2000)	oligophage	broad	(Noyes, 2014)	adults	17.30	(Couty et al., 2001)	solitary
Aphelinus asychis (Walker)	learned	.	(De Farias and Hopper, 1997)	oligophage	broad	(Noyes, 2014)	adults	19.70	(Jackson and Eikenbary, 1971)	solitary
Encarsia formosa (Gahan)	learned	.	(Guerrieri, 1997; Perez-Hedo et al., 2015)	specialist	broad	(Thompson, 1953)	larvae	52.00	(Jervis and Ferns, 2011)	solitary
**Hymenoptera: Encyrtidae**										
Epidinocarsis lopezi (De Santis)	innate	.	(Souissi et al., 1998)	specialist	narrow	(Noyes, 2014)	larvae	41.40	(Iziquel and Le Rü, 1992)	solitary
**Hymenoptera: Eulophidae**										
Closterocerus ruforum (Krausse)	learned	specific	(Hilker et al., 2002; Mumm et al., 2005)	specialist	narrow	(Thompson, 1955)	eggs	.	.	solitary
Diglyphus isaea (Walker)	.	generic	(Finidori-Logli et al., 1996)	generalist	broad	(Noyes, 2014)	larvae	21.00	(Jervis and Ferns, 2011)	solitary
Hyssopus pallidus (Askew)	innate	specific	(Mattiacci et al., 1999)	oligophage	broad	(Zaviezo and Mills, 1999)	larvae	19.00	(Jervis and Ferns, 2011)	gregarious
Oomyzus gallerucae (Fonscolombe)	innate	specific	(Meiners and Hilker, 1997; Meiners et al., 2000; Büchel et al., 2011)	specialist	narrow	(Thompson, 1955)	eggs	.	.	solitary
**Hymenoptera: Myrmaridae**										
Anagrus breviphragma (Soyka)	innate	.	(Chiappini et al., 2012)	generalist	broad	(Chiappini et al., 2015)	eggs	4.98	(Virla, 2001)	solitary
Anagrus nilaparvatae (Pang & Wang)	innate	specific	(Lou et al., 2005)	specialist	narrow	(Yean and Xiongfei, 1986)	eggs	1.19	(Zhu et al., 2013)	solitary
Anaphes iole (Girault)	learned	specific	(Manrique et al., 2005)	specialist	narrow	(Thompson, 1958)	eggs	10.80	(Jervis and Ferns, 2011)	solitary
**Hymenoptera: Pteromalidae**										
Lariophagus distinguendus (Foster)	innate	specific	(Steidle and Schöller, 1997)	generalist	broad	(Noyes, 2014)	larvae	39.73	(Jervis and Ferns, 2011)	solitary
Rhopalicus tutela (Walker)	innate	.	(Pettersson, 2001)	generalist	narrow	(Noyes, 2014)	larvae	.	.	solitary
Roptrocerus mirus (Walker)	innate	.	(Pettersson, 2001)	oligophage	narrow	(Noyes, 2014)	larvae	.	.	solitary
Roptrocerus xylophagorum (Ratzeburg)	.	specific	(Sullivan et al., 2000)	oligophage	narrow	(Thompson, 1958)	larvae	7.90	(Samson, 1984)	solitary
**Hymenoptera: Trichogrammatidae**										
Trichogramma bournieri (Pintureau)	innate	.	(Tamiru et al., 2011)	generalist	narrow	(Noyes, 2014)	eggs	6.17	(Haile et al., 2002)	solitary
Trichogramma brassicae (Bezdenko)	innate	.	(Cusumano et al., 2015)	generalist	broad	(Noyes, 2014)	eggs	11.70	(Bjorksten and Hoffmann, 1998)	solitary
Trichogramma chilonis (Ishii)	innate	generic	(Raghava et al., 2010)	generalist	broad	(Noyes, 2014)	eggs	6.00	(Miura and Kobayashi, 1995)	gregarious
Trichogramma evanescens (Westwood)	innate	.	(Cusumano et al., 2015)	generalist	broad	(Noyes, 2014)	eggs	4.76	(Doyon and Boivin, 2005)	gregarious
Trichogramma japonicum (Ashmead)	innate	.	(Rani and Sandhyarani, 2012)	generalist	broad	(Noyes, 2014)	eggs	.	.	gregarious
Trichogramma pretiosum (Riley)	innate	.	(Peñaflor et al., 2011)	generalist	broad	(Noyes, 2014)	eggs	13.90	(Brotodjojo and Walter, 2006)	gregarious
**Hymenoptera: Figitidae**										
Trybliographa rapae (Westwood)	innate	specific	(Neveu et al., 2002)	specialist	narrow	(Wishart and Monteith, 1954)	larvae	14.40	(Jervis and Ferns, 2011)	solitary
**Hymenoptera: Braconidae**										
Aphidius colemani (Viereck)	learned	.	(Grasswitz, 1998)	specialist	broad	(Messing and Rabasse, 1995)	adults	12.00	(Hofsvang and Hågvar, 1975)	solitary
Aphidius ervi (Haliday)	innate	specific	(Du et al., 1996)	specialist	broad	(Thompson, 1953)	adults	15.40	(Hofsvang and Hågvar, 1975)	solitary
Aphidius funebris (Mackauer)	innate	.	(Pareja et al., 2007)	specialist	broad	(Thompson, 1953)	adults	.	.	solitary
Aphidius matricariae (Haliday)	innate	.	(De Farias and Hopper, 1997)	specialist	broad	(Thompson, 1953)	adults	23.80	(Reed et al., 1992)	solitary
Binodoxys communis (Gahan)	learned	specific	(Wyckhuys and Heimpel, 2007)	specialist	broad	(Thompson, 1953)	adults	6.00	(Wyckhuys et al., 2008)	solitary

Cotesia flavipes (Cameron)	innate	specific	(Potting et al., 1995)	generalist	broad	(Thompson, 1953)	larvae	7.90	(Jervis and Ferns, 2011)	gregarious
Cotesia glomerata (Linnaeus)	innate	generic	(Mattiacci et al., 1994; Geervliet et al., 1996; Connor et al., 2007)	generalist	broad	(Thompson, 1953)	larvae	12.50	(Lee and Heimpel, 2008)	gregarious
Cotesia kariyai (Watanebe)	innate	specific	(Takabayashi et al., 1995)	specialist	narrow	(Fujiwara et al., 2000)	larvae	.	.	gregarious
Cotesia marginiventris (Cresson)	innate	generic	(Turlings et al., 1993; Hoballah and Turlings, 2005)	generalist	broad	(Thompson, 1953)	larvae	18.10	(Jervis and Ferns, 2011)	solitary
Cotesia rubecula (Marshall)	innate	generic	(Van Poecke et al., 2001)	specialist	narrow	(Thompson, 1953)	larvae	16.50	(Sengonca and Peters, 1993)	solitary
Cotesia sesamiae (Cameron)	innate	.	(Ngi-Song et al., 1996; Bruce et al., 2010)	generalist	narrow	(Thompson, 1953)	larvae	.	.	gregarious
Cotesia vestalis (Haliday)	innate	specific	(Shiojiri et al., 2000; Girling et al., 2011)	specialist	narrow	(Thompson, 1953)	larvae	5.00	(Jervis and Ferns, 2011)	solitary
Diachasmimorpha juglandis (Muesebeck)	innate	generic	(Henneman et al., 2002)	specialist	narrow	(Wharton and Marsh, 1978)	larvae	.	.	solitary
Diachasmimorpha krausii (Fullaway)	innate	.	(Ero and Clarke, 2012)	specialist	broad	(Ero, 2009)	larvae	22.50	(Sime et al., 2006)	solitary
Diachasmimorpha longicaudata (Ashmead)	innate	specific	(Carrasco et al., 2005)	oligophage	broad	(Wharton and Marsh, 1978)	larvae	27.00	(Sime et al., 2006)	solitary
Diaeretiella rapae (M'Intosh)	learned	specific	(Girling et al., 2006; Agbogba and Powell, 2007)	specialist	broad	(Thompson, 1953)	adults	19.70	(Reed et al., 1992)	solitary
Fopius arisanus (Sonan)	innate	specific	(Pérez et al., 2013)	oligophage	broad	(Rousse et al., 2005)	eggs	23.23	(Jervis and Ferns, 2011)	solitary
Glyptapanteles flavicoxis (Marsh)	innate	.	(Havill and Raffa, 2000)	specialist	broad	(Hopper, 2003)	larvae	.	.	gregarious
Glyptapanteles liparidis (Bouch?)	innate	.	(McCormick et al., 2014)	generalist	broad	(Hopper, 2003)	larvae	15.50	(Tillinger et al., 2004)	gregarious
Microplitis croceipes (Cresson)	learned	generic	(McCall et al., 1993; Turlings et al., 1993)	specialist	broad	(Thompson, 1953)	larvae	28.00	(Jervis and Ferns, 2011)	solitary
Microplitis mediator (Haliday)	innate	specific	(Yu et al., 2010; Pangesti et al., 2015)	generalist	broad	(Yu, 2012)	larvae	44.00	(Luo et al., 2010)	solitary
Microplitis rufiventris (Kok)	innate	generic	(Gouinguene et al., 2003; Hoballah and Turlings, 2005)	oligophage	broad	(Tamo et al., 2006)	larvae	9.95	(Jervis and Ferns, 2011)	solitary
Opius dissitus (Muesebeck)	innate	.	(Petitt et al., 1992)	specialist	broad	(Johnson and Hara, 1987)	larvae	.	.	solitary
Orgilus lepidus (Muesebeck)	.	generic	(Keller and Horne, 1993)	specialist	narrow	(Yu, 2012)	larvae	21.40	(Oatman et al., 1969)	solitary
Pholetesor bicolor (Ness)	innate	.	(Dutton et al., 2000)	generalist	broad	(Thompson, 1953)	larvae	.	.	solitary
Toxoneuron nigriceps (Viereck)	innate	specific	(De Moraes et al., 1998)	specialist	broad	(Thompson, 1953)	larvae	28.00	(Butler et al., 1983)	solitary
**Hymenoptera: Ichneumonidae**										
Campoletis chlorideae (Uchida)	innate	generic	(Yan et al., 2005)	generalist	broad	(Yu, 2012)	larvae	17.20	(Pandey and Tripathi, 2008)	solitary
Campoletis flavicincta (Ashmead)	innate	specific	(Signoretti et al., 2012)	generalist	broad	(Marino et al., 2006)	larvae	14.60	(Zanuncio et al., 2013)	solitary
Campoletis sonorensis (Cameron)	innate	.	(Tamo et al., 2006)	generalist	broad	(Yu, 2012)	larvae	14.00	(Sanders et al., 2007)	solitary
Diadegma fenestrale (Holmgren)	innate	generic	(Gols et al., 2012)	generalist	broad	(Thompson, 1957)	larvae	.	.	solitary
Diadegma semiclausum (Hellen)	innate	generic	(Gols et al., 2012)	generalist	broad	(Bartlett and Clausen, 1978)	larvae	18.40	(Jervis and Ferns, 2011)	solitary
Diadromus pulchellus (Wesmael)	innate	specific	(Dugravot and Thibout, 2006)	specialist	narrow	(Jenner, 2008)	larvae	43.60	(Jenner et al., 2012)	solitary
Glypta haesitator (Gravenhorst)	innate	.	(Dalen et al., 2015)	generalist	broad	(Dalen, 2012)	larvae	30.00	(Dalen, 2012)	solitary
Dentichasmias busseolae (Heinrich)	innate	.	(Gohole et al., 2003)	generalist	narrow	(Muyekho et al., 2005)	larvae	.	.	solitary
**Hymenoptera: Scelionidae**										
Telenomus busseolae (Gahan)	innate	.	(Salerno et al., 2013)	generalist	broad	(Fantinou et al., 1998)	eggs	21.70	(Chabi-Olaye et al., 1997)	solitary
Telenomus podisi (Ashmead)	innate	.	(Moraes et al., 2005)	generalist	broad	(Thompson, 1958)	eggs	12.20	(Yeargan, 1982)	solitary
Telenomus remus (Nixon)	learned	.	(Peñaflor et al., 2011)	generalist	broad	(Wojcik et al., 1976)	eggs	18.00	(Schwartz and Gerling, 1974)	solitary
Trissolcus basalis (Wollaston)	innate	specific	(Colazza et al., 2004; Moujahed et al., 2014)	specialist	broad	(Thompson, 1958)	eggs	33.35	(Awan et al., 1990)	solitary
Trissolcus brochymenae (Ashmead)	innate	generic	(Conti et al., 2010; Frati et al., 2013)	oligophage	broad	(Torres et al., 2002)	eggs	17.60	(Torres et al., 2002)	solitary
**Hymenoptera: Tiphiidae**										
Tiphia popilliavora (Rohwer)	innate	.	(Obeysekara et al., 2014)	specialist	broad	(Obeysekara, 2013)	larvae	22.77	(Holloway, 1931)	solitary
Tiphia vernalis (Rohwer)	innate	.	(Obeysekara et al., 2014)	specialist	broad	(Obeysekara, 2013)	larvae	18.60	(Rogers and Potter, 2003)	solitary
**Diptera: Tachinidae**										
Exorista japonica (Townsend)	innate	specific	(Ichiki et al., 2008)	specialist	broad	(Thompson, 1951)	larvae	34.20	(Nakamura, 1994)	gregarious
Pales pavida (Meigen)	innate	specific	(Ichiki et al., 2012)	generalist	broad	(Thompson, 1951)	larvae	10.50	(Hirose, 2005)	gregarious
Zenillia dolosa (Meigen)	innate	generic	(Ichiki et al., 2012)	generalist	broad	(Ho et al., 2011)	larvae	22.70	(Ho et al., 2011)	gregarious

B Phylogenetic tree

Phylogenetic correlations among the different traits (response specificity, response innateness, and the life-history traits) were quantified (Table B.2) using Abouheif's test (Pavoine et al., 2008). This approach measures Moran's I based on a matrix of phylogetic proximities and performs independent Monte Carlo tests (999 permutations) for each trait (Jombart et al., 2010).

To remove the effects of phylogenetic autocorrelation, we used autoregressive models (Cheverud et al., 1985) by including a lag vector in the generalized linear model. The lag vector $\bar{x}_i$ was defined for each response category x_i, i $\in$ $\{$innateness, specificity $\}$, such that $\bar{x}_i = Wx$, where W was the Abouheif's matrix of phylogenetic proximities (Pavoine et al., 2008).

Table B.2: Estimates of phylogenetic signals

**Traits**	**Observed Moran's I**	**p-value**
Response specificity	-0.07	0.71
Response innateness	0.19	0.02
Host specialization	0.25	0.003
Host dietary breadth	0.22	0.005
Target host life stage	0.68	0.001
Lifespan	0.14	0.05
Egg-laying pattern	0.44	0.001

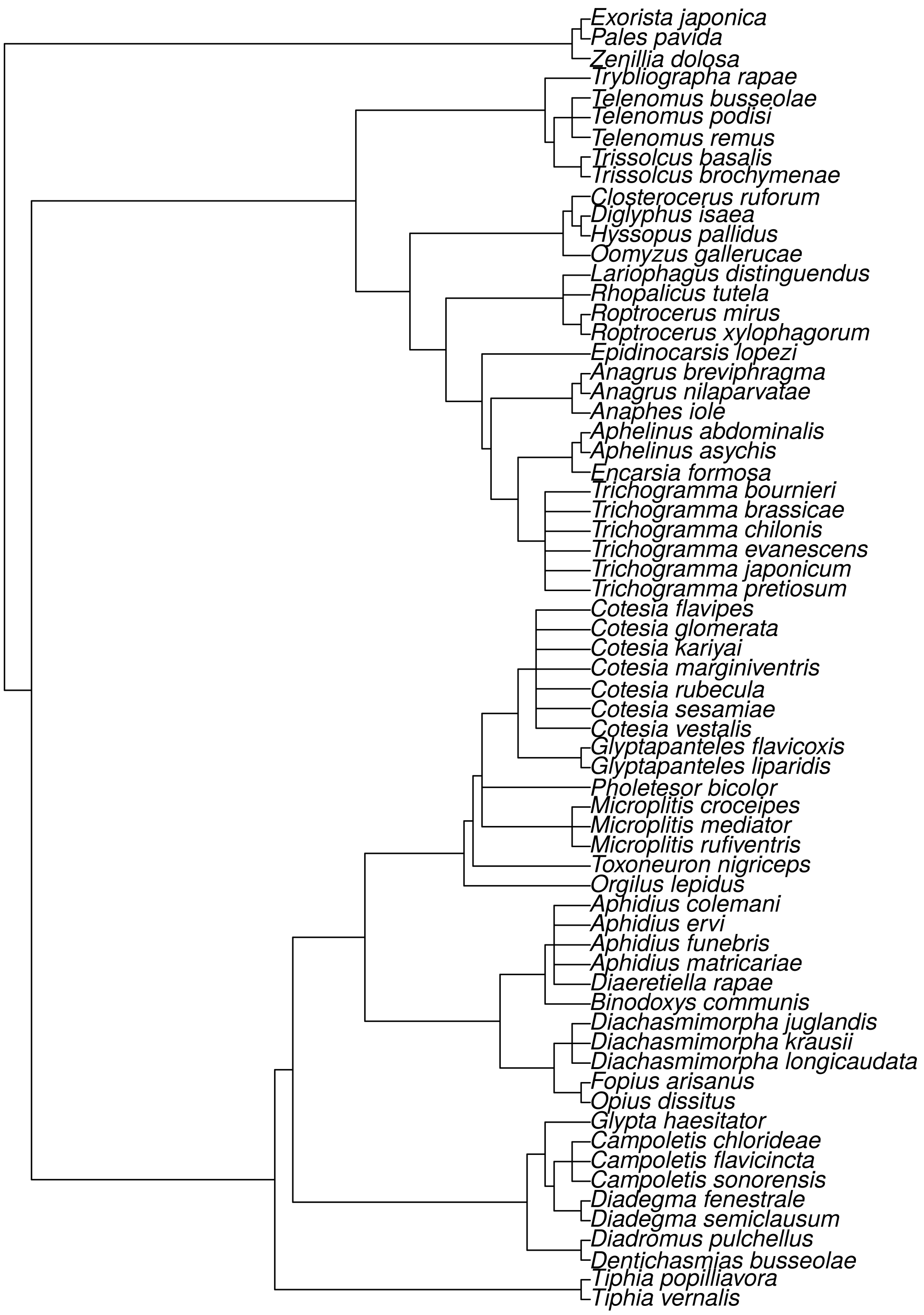

Figure B.1: Phylogenetic tree of the 66 study species

C Parasitoid response to HIPVs

C.1 Response specificity

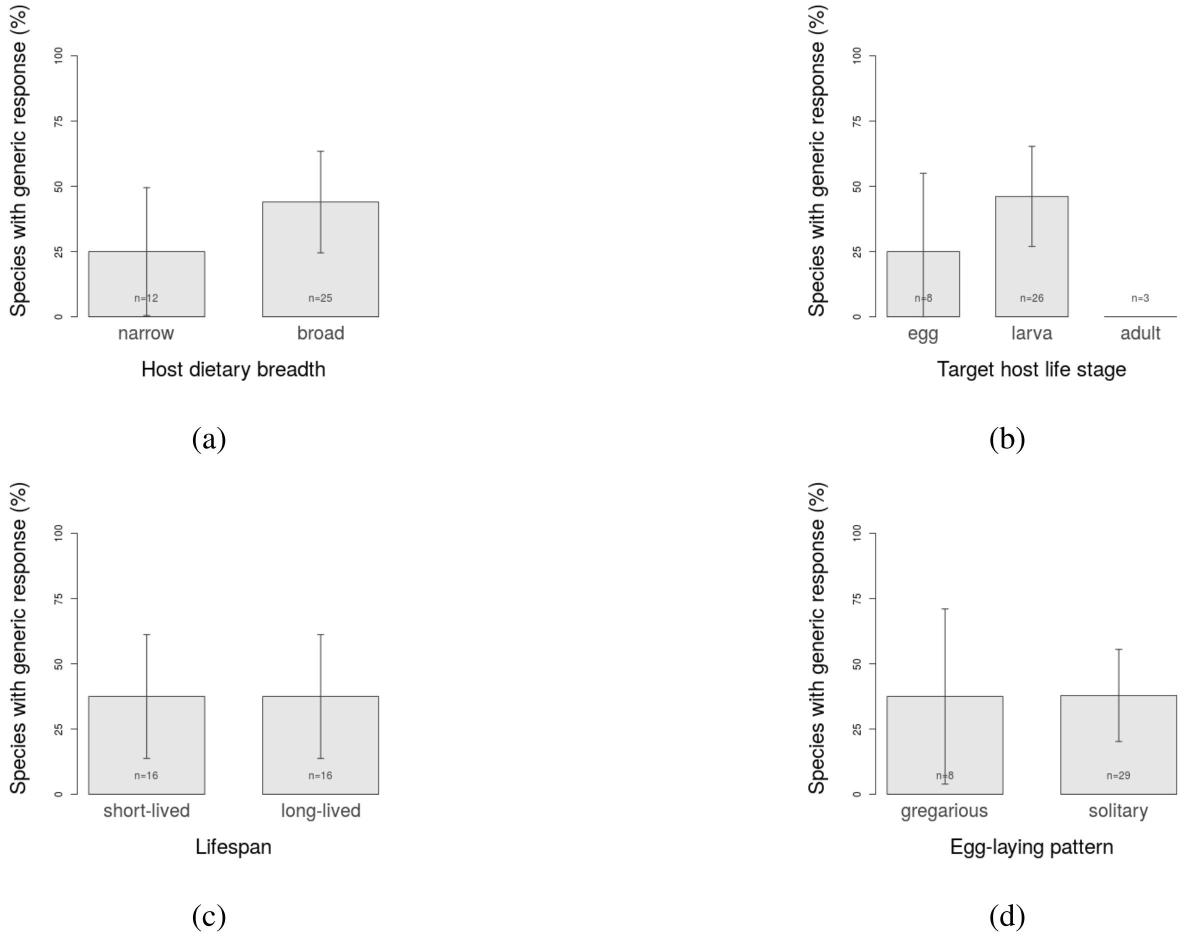

Figure C.1: Percentage of species displaying generic responses according to (a) host dietary breadth, (b) target host life stage, (c) lifespan (transformed into a two-category variable), and (d) egg-laying pattern. The bars represent the observed percentages with 95% confidence intervals (n is specified for each category).

References

Agbogba, B. C., and W. Powell. 2007. Effect of the presence of a nonhost herbivore on the response of the aphid parasitoid *Diaeretiella rapae* to host-infested cabbage plants. Journal of Chemical Ecology 33:2229–2235.

Awan, M. S., L. T. Wilson, and M. P. Hoffmann. 1990. Comparative biology of three geographic populations of *Trissolcus basalis* (Hymenoptera: Scelionidae). Environmental Entomology 19:387–392.

Bartlett, B. R., and C. P. Clausen. 1978. Introduced parasites and predators of arthropod pests and weeds: a world review. Washington, D.C.: Agricultural Research Service, U.S. Dept. of Agriculture.

Bjorksten, T. A., and A. A. Hoffmann. 1998. Persistence of experience effects in the parasitoid *Trichogramma* nr. *brassicae*. Ecological Entomology 23:110–117.

Brotodjojo, R. R. R., and G. H. Walter. 2006. Oviposition and reproductive performance of a generalist parasitoid (*Trichogramma pretiosum*) exposed to host species that differ in their physical characteristics. Biological Control 39:300–312.

Bruce, T. J., C. A. Midega, M. A. Birkett, J. A. Pickett, and Z. R. Khan. 2010. Is quality more important than quantity? Insect behavioural responses to changes in a volatile blend after stemborer oviposition on an African grass. Biology letters 6:314–317.

Büchel, K., S. Malskies, M. Mayer, T. M. Fenning, J. Gershenzon, M. Hilker, and T. Meiners. 2011. How plants give early herbivore alert: volatile terpenoids attract parasitoids to egg-infested elms. Basic and Applied Ecology 12:403–412.

Butler, G. D., A. G. Hamilton, and J. D. Lopez. 1983. *Cardiochiles nigriceps* (Hymenoptera: Braconidae): development time and fecundity in relation to temperature. Annals of the Entomological Society of America 76:536–538.

Carrasco, M., P. Montoya, L. Cruz-Lopez, and J. C. Rojas. 2005. Response of the fruit fly parasitoid *Diachasmimorpha longicaudata* (Hymenoptera: Braconidae) to mango fruit volatiles. Environmental Entomology 34:576–583.

Chabi-Olaye, A., F. Schulthess, T. Shanower, and N. Bosque-Perez. 1997. Factors Influencing the Developmental Rates and Reproductive Potentials of *Telenomus busseolae* (Gahan)[Hym.: Scelionidae], an Egg Parasitoid of *Sesamia calamistis* Hampson (Lep.: Noctuidae). Biological Control 8:15–21.

Cheverud, J. M., M. M. Dow, and W. Leutenegger. 1985. The quantitative assessment of phylogenetic constraints in comparative analyses: sexual dimorphism in body weight among primates. Evolution 39:1335–1351.

Chiappini, E., A. Berzolla, and A. Oppo. 2015. *Anagrus breviphragma* soyka short distance search stimuli. BioMed Research International 2015.

Chiappini, E., G. Salerno, A. Berzolla, A. Iacovone, M. Cristina Reguzzi, and E. Conti. 2012. Role of volatile semiochemicals in host location by the egg parasitoid *Anagrus breviphragma*. Entomologia Experimentalis et Applicata 144:311–316.

Colazza, S., J. McElfresh, and J. Millar. 2004. Identification of volatile synomones, induced by *Nezara viridula* feeding and oviposition on bean spp., that attract the egg parasitoid *Trissolcus basalis*. Journal of Chemical Ecology 30:945–964.

Connor, E. C., A. S. Rott, J. Samietz, and S. Dorn. 2007. The role of the plant in attracting parasitoids: response to progressive mechanical wounding. Entomologia Experimentalis et Applicata 125:145–155.

Conti, E., G. Salerno, B. Leombruni, F. Frati, and F. Bin. 2010. Short-range allelochemicals from a plant-herbivore association: a singular case of oviposition-induced synomone for an egg parasitoid. Journal of Experimental Biology 213:3911–3919.

Couty, A., S. J. Clark, and G. M. Poppy. 2001. Are fecundity and longevity of female *Aphelinus abdominalis* affected by development in GNA-dosed *Macrosiphum euphorbiae*? Physiological Entomology 26:287–293.

Cusumano, A., B. T. Weldegergis, S. Colazza, M. Dicke, and N. E. Fatouros. 2015. Attraction of egg-killing parasitoids toward induced plant volatiles in a multi-herbivore context. Oecologia 179:163–174.

Dalen, M. 2012. *Glypta haesitator* host location. M.S. thesis. Norwegian University of Life Sciences.

Dalen, M., G. K. Knudsen, H. R. Norli, and G. Thoming. 2015. Sources of volatiles mediating host location behaviour of *Glypta haesitator*, a larval parasitoid of *Cydia nigricana*. Biological Control 90:128–140.

De Farias, A. M. I., and K. R. Hopper. 1997. Responses of female *Aphelinus asychis* (Hymenoptera: Aphelinidae) and *Aphidius matricariae* (Hymenoptera: Aphidiidae) to host and plant-host odors. Environmental Entomology 26:989–994.

De Moraes, C. M., W. J. Lewis, P. W. Pare, H. T. Alborn, and J. H. Tumlinson. 1998. Herbivore-infested plants selectively attract parasitoids. Nature 393:570–573.

Doyon, J., and G. Boivin. 2005. The effect of development time on the fitness of female *Trichogramma evanescens*. Journal of Insect Science 5:4.

Du, Y., G. Poppy, and W. Powell. 1996. Relative importance of semiochemicals from first and second trophic levels in host foraging behavior of *Aphidius ervi*. Journal of Chemical Ecology 22:1591–1605.

Dugravot, S., and E. Thibout. 2006. Consequences for a specialist insect and its parasitoid of the response of *Allium porrum* to conspecific herbivore attack. Physiological Entomology 31:73–79.

Dutton, A., L. Mattiacci, and S. Dorn. 2000. Plant-derived semiochemicals as contact host location stimuli for a parasitoid of leafminers. Journal of Chemical Ecology 26:2259–2273.

Ero, M. M. 2009. Host searching behaviour of *Diachasmimorpha kraussii* (Fullaway) (Hymenopetra: Braconidae: Opiinae), a polyphagous parasitoid of Dacinae fruit flies (Diptera: Tephritidae). Ph.D. thesis. Queensland University of Technology.

Ero, M. M., and A. R. Clarke. 2012. Host location by the fruit fly parasitoid *Diachasmimorpha krausii*: role of fruit fly species, life stage and host plant. Agricultural and Forest Entomology 14:101–110.

Fantinou, A. A., M. P. Alexandri, and J. A. Tsitsipis. 1998. Adult emergence rhythm of the egg-parasitoid *Telenomus busseolae*. Biocontrol 43:141–151.

Finidori-Logli, V., A.-G. Bagnères, and J.-L. Clément. 1996. Role of plant volatiles in the search for a host by parasitoid *Diglyphus isaea* (Hymenoptera: Eulophidae). Journal of Chemical Ecology 22:541–558.

Frati, F., G. Salerno, and E. Conti. 2013. Cabbage waxes affect *Trissolcus brochymenae* response to short-range synomones. Insect Science 20:753–762.

Fujiwara, C., J. Takabayashi, and S. Yano. 2000. Effects of host-food plant species on parasitization rates of *Mythimna separata* (Lepidoptera: Noctuidae) by a parasitoid, *Cotesia kariyai* (Hymenoptera: Braconidae). Applied Entomology and Zoology 35:131–136.

Geervliet, J. B., L. E. Vet, and M. Dicke. 1996. Innate responses of the parasitoids *Cotesia glomerata* and *C. rubecula* (Hymenoptera: Braconidae) to volatiles from different plant-herbivore complexes. Journal of Insect Behavior 9:525–538.

Girling, R. D., M. Hassall, J. G. Turner, and G. M. Poppy. 2006. Behavioural responses of the aphid parasitoid *Diaeretiella rapae* to volatiles from *Arabidopsis thaliana* induced by *Myzus persicae*. Entomologia Experimentalis et Applicata 120:1–9.

Girling, R. D., A. Stewart-Jones, J. Dherbecourt, J. T. Staley, D. J. Wright, and G. M. Poppy. 2011. Parasitoids select plants more heavily infested with their caterpillar hosts: a new approach to aid interpretation of plant headspace volatiles. Proceedings of the Royal Society B-biological Sciences 278:2646–2653.

Gohole, L. S., W. A. Overholt, Z. R. Khan, and L. E. Vet. 2003. Role of volatiles emitted by host and non-host plants in the foraging behaviour of *Dentichasmias busseolae*, a pupal parasitoid of the spotted stemborer *Chilo partellus*. Entomologia Experimentalis et Applicata 107:1–9.

Gols, R., C. Veenemans, R. P. J. Potting, H. M. Smid, M. Dicke, J. A. Harvey, and T. Bukovinszky. 2012. Variation in the specificity of plant volatiles and their use by a specialist and a generalist parasitoid. Animal Behaviour 83:1231–1242.

Gouinguene, S., H. Alborn, and T. C. J. Turlings. 2003. Induction of volatile emissions in maize by different larval instars of *Spodoptera littoralis*. Journal of Chemical Ecology 29:145–162.

Grasswitz, T. R. 1998. Effect of adult experience on the host-location behavior of the aphid parasitoid *Aphidius colemani* Viereck (Hymenoptera: Aphidiidae). Biological Control 12:177–181.

Guerrieri, E. 1997. Flight behaviour of *Encarsia formosa* in response to plant and host stimuli. Entomologia Experimentalis et Applicata 82:129–133.

Haile, A. T., S. A. Hassan, S. Sithanantham, C. K. P. O. Ogol, and J. Baumgärtner. 2002. Comparative life table analysis of *Trichogramma bournieri* Pintureau and Babault and *Trichogramma* sp. nr. mwanzai Schulten and Feijen (Hym., Trichogrammatidae) from Kenya. Journal of Applied Entomology 126:287–292.

Havill, N. P., and K. F. Raffa. 2000. Compound effects of induced plant responses on insect herbivores and parasitoids: implications for tritrophic interactions. Ecological Entomology 25:171–179.

Henneman, M. L., E. G. Dyreson, J. Takabayashi, and R. A. Raguso. 2002. Response to walnut olfactory and visual cues by the parasitic wasp *Diachasmimorpha juglandis*. Journal of Chemical Ecology 28:2221–2244.

Hilker, M., C. Kobs, M. Varma, and K. Schrank. 2002. Insect egg deposition induces *Pinus sylvestris* to attract egg parasitoids. Journal of Experimental Biology 205:455–461.

Hirose, Y. 2005. Discovery of insect parasitism and subsequent development of parasitoid research in Japan. Biological Control 32:49–56.

Ho, G. T., R. T. Ichiki, and S. Nakamura. 2011. Reproductive biology of the microtype tachinid fly *Zenillia dolosa* (Meigen)(Diptera: Tachinidae). Entomological Science 14:210–215.

Hoballah, M. E., and T. C. J. Turlings. 2005. The role of fresh versus old leaf damage in the attraction of parasitic wasps to herbivore-induced maize volatiles. Journal of Chemical Ecology 31:2003–2018.

Hofsvang, T., and E. B. Hågvar. 1975. Duration of development and longevity in *Aphidius ervi* and *Aphidius platensis* [Hym.: Aphidiidae], two parasites of *Myzus persicae* [Hym.: Aphididae]. Entomophaga 20:11–22.

Holloway, J. K. 1931. Temperature as a factor in the activity and development of the Chinese strain of *Tiphia popilliavora* (Rohw.) in New Jersey and Pennsylvania. Journal of the New York Entomological Society pages 555–565.

Hopper, K. R. 2003. Biological control:benefits and risks, chap. Potential impacts on threatened and endangered insect species in the United States from introductions of parasitic Hymenoptera for the control of insect pests, pages 64–74. Cambridge University Press.

Ichiki, R. T., G. T. T. Ho, E. Wajnberg, Y. Kainoh, J. Tabata, and S. Nakamura. 2012. Different uses of plant semiochemicals in host location strategies of the two tachinid parasitoids. Naturwissenschaften 99:687–694.

Ichiki, R. T., Y. Kainoh, S. Kugimiya, J. Takabayashi, and S. Nakamura. 2008. Attraction to herbivore-induced plant volatiles by the host-foraging parasitoid fly *Exorista japonica*. Journal of Chemical Ecology 34:614–621.

Iziquel, Y., and B. Le Rü. 1992. Fecundity, longevity, and intrinsic natural rate of increase of *Epidinocarsis lopezi* (de santis)(Hymenoptera: Encyrtidae). The Canadian Entomologist 124:1115–1121.

Jackson, H. B., and R. D. Eikenbary. 1971. Bionomics of *Aphelinus asychis* (Hymenoptera: Eulophidae) an introduced parasite of the sorghum greenbug. Annals of the Entomological Society of America 64:81–85.

Jenner, W. H. 2008. Evaluation of a candidate classical biological control agent and critical assessment of suggested host specificity testing guidelines. Ph.D. thesis. Carleton University.

Jenner, W. H., U. Kuhlmann, N. Cappuccino, and P. G. Mason. 2012. Manipulation of parasitoid state influences host exploitation by *Diadromus pulchellus* Wesmael (Hymenoptera: Ichneumonidae). Biological Control 63:264–269.

Jervis, M., and P. Ferns. 2011. Towards a general perspective on life-history evolution and diversification in parasitoid wasps. Biological Journal of the Linnean Society 104:443–461.

Johnson, M. W., and A. H. Hara. 1987. Influence of host crop on parasitoids (Hymenoptera) of *Liriomyza* spp.(Diptera: Agromyzidae). Environmental entomology 16:339–344.

Jombart, T., F. Balloux, and S. Dray. 2010. Adephylo: new tools for investigating the phylogenetic signal in biological traits. Bioinformatics 26:1907–1909.

Keller, M. A., and P. A. Horne. 1993. Sources of host-location cues for the parasitic wasp *Orgilus lepidus* (Braconidae). Australian Journal of Zoology 41:335–341.

Lee, J. C., and G. E. Heimpel. 2008. Effect of floral nectar, water, and feeding frequency on *Cotesia glomerata* longevity. BioControl 53:289–294.

Lou, Y. G., B. Ma, and J. A. Cheng. 2005. Attraction of the parasitoid *Anagrus nilaparvatae* to rice volatiles induced by the rice brown planthopper *Nilaparvata lugens*. Journal of Chemical Ecology 31:2357–2372.

Luo, S., J. Li, X. Liu, Z. Lu, W. Pan, Q. Zhang, and Z. Zhao. 2010. Effects of six sugars on the longevity, fecundity and nutrient reserves of *Microplitis mediator*. Biological Control 52:51–57.

Manrique, V., W. A. Jones, L. H. Williams III, and J. S. Bernal. 2005. Olfactory responses of *Anaphes iole* (Hymenoptera: Mymaridae) to volatile signals derived from host habitats. Journal of Insect Behavior 18:89–104.

Marino, P. C., D. A. Landis, and B. A. Hawkins. 2006. Conserving parasitoid assemblages of north american pest lepidoptera: Does biological control by native parasitoids depend on landscape complexity? Biological Control 37:173–185.

Mattiacci, L., M. Dicke, and M. A. Posthumus. 1994. Induction of parasitoid attracting synomone in brussels-sprouts plants by feeding of *Pieris brassicae* larvae - role of mechanical damage and herbivore elicitor. Journal of Chemical Ecology 20:2229–2247.

Mattiacci, L., E. Hütter, and S. Dorn. 1999. Host location of *Hyssopus pallidus*, a larval parasitoid of the codling moth, *Cydia pomonella*. Biological Control 15:241 – 251.

McCall, P. J., T. C. J. Turlings, W. J. Lewis, and J. H. Tumlinson. 1993. Role of plant volatiles in host location by the specialist parasitoid *Microplitis croceipes* Cresson (Braconidae: Hymenoptera). Journal of Insect Behavior 6:625–639.

McCormick, A. C., S. Irmisch, A. Reinecke, G. A. Boeckler, D. Veit, M. Reichelt, B. S. Hansson, J. Gershenzon, T. G. Koellner, and S. B. Unsicker. 2014. Herbivore-induced volatile emission in black poplar: regulation and role in attracting herbivore enemies. Plant Cell and Environment 37:1909–1923.

Meiners, T., and M. Hilker. 1997. Host location in *Oomyzus gallerucae* (Hymenoptera: Eulophidae), an egg parasitoid of the elm leaf beetle *Xanthogaleruca luteola* (Coleoptera: Chrysomelidae). Oecologia 112:87–93.

Meiners, T., C. Westerhaus, and M. Hilker. 2000. Specificity of chemical cues used by a specialist egg parasitoid during host location. Entomologia Experimentalis et Applicata 95:151–159.

Messing, R., and J. Rabasse. 1995. Oviposition behaviour of the polyphagous aphid parasitoid *Aphidius colemani* Viereck (Hymenoptera: Aphidiidae). Agriculture, Ecosystems & Environment 52:13–17.

Miura, K., and M. Kobayashi. 1995. Reproductive properties of *Trichogramma chilonis* females on diamondback moth eggs. Applied Entomology and Zoology 30:393–393.

Molck, G., H. Pinn, and U. Wyss. 2000. Manipulation of plant odour preference by learning in the aphid parasitoid *Aphelinus abdominalis* (Hymenoptera: Aphelinidae). European Journal of Entomology 97:533–538.

Moraes, M. C. B., R. Laumann, E. R. Sujii, C. Pires, and M. Borges. 2005. Induced volatiles in soybean and pigeon pea plants artificially infested with the neotropical brown stink bug, *Euschistus heros*, and their effect on the egg parasitoid, *Telenomus podisi*. Entomologia Experimentalis et Applicata 115:227–237.

Moujahed, R., F. Frati, A. Cusumano, G. Salerno, E. Conti, E. Peri, and S. Colazza. 2014. Egg parasitoid attraction toward induced plant volatiles is disrupted by a non-host herbivore attacking above or belowground plant organs. Frontiers in Plant Science 5:601.

Mumm, R., T. Tiemann, M. Varama, and M. Hilker. 2005. Choosy egg parasitoids: specificity of oviposition-induced pine volatiles exploited by an egg parasitoid of pine sawflies. Entomologia Experimentalis et Applicata 115:217–225.

Muyekho, F. N., A. T. Barrion, and Z. R. Khan. 2005. Host range for stemborers and associated natural enemies in different farming systems of Kenya. African Crop Science Journal 13.

Nakamura, S. 1994. Parasitization and life history parameters of *Exorista japonica* (Diptera: Tachinidae) using the common armyworm, *Pseudaletia separata* (Lepidoptera: Noctuidae) as a host. Applied Entomology and Zoology 29:133–140.

Neveu, N., J. Grandgirard, J. P. Nenon, and A. M. Cortesero. 2002. Systemic release of herbivore-induced plant volatiles by turnips infested by concealed root-feeding larvae *Delia radicum* L. Journal of Chemical Ecology 28:1717–1732.

Ngi-Song, A. J., W. A. Overholt, P. G. N. Njagi, M. Dicke, J. N. Ayertey, and W. Lwande. 1996. Volatile infochemicals used in host and host habitat location by *Cotesia flavipes* Cameron and

Cotesia sesamiae (Cameron)(Hymenoptera: Braconidae), larval parasitoids of stemborers on graminae. Journal of Chemical Ecology 22:307–323.

Noyes, J. 2014. Universal Chalcidoidea Database. http://www.nhm.ac.uk/chalcidoids.

Oatman, E. R., G. R. Platner, and P. D. Greany. 1969. The biology of *Orgilus lepidus* (Hymenoptera: Braconidae), a primary parasite of the potato tuberworm. Annals of the Entomological Society of America 62:1407–1414.

Obeysekara, P. T. 2013. Host selection of spring Tiphia (*Tiphia vernalis*) and summer Tiphia (*Tiphia popilliavora*), natural enemies of Japanese and oriental beetles. Ph.D. thesis. University of Connecticut,.

Obeysekara, P. T., A. Legrand, and G. Lavigne. 2014. Use of herbivore-induced plant volatiles as search cues by *Tiphia vernalis* and *Tiphia popilliavora* to locate their below-ground scarabaeid hosts. Entomologia Experimentalis et Applicata 150:74–85.

Pandey, A. K., and C. Tripathi. 2008. Effect of temperature on the development, fecundity, progeny sex ratio and life-table of *Campoletis chlorideae*, an endolarval parasitoid of the pod borer, *Helicoverpa armigera*. BioControl 53:461–471.

Pangesti, N., B. T. Weldegergis, B. Langendorf, J. J. A. van Loon, M. Dicke, and A. Pineda. 2015. Rhizobacterial colonization of roots modulates plant volatile emission and enhances the attraction of a parasitoid wasp to host-infested plants. Oecologia 178:1169–1180.

Pareja, M., M. C. Moraes, S. J. Clark, M. A. Birkett, and W. Powell. 2007. Response of the aphid parasitoid *Aphidius funebris* to volatiles from undamaged and aphid-infested *Centaurea nigra*. Journal of Chemical Ecology 33:695–710.

Pavoine, S., S. Ollier, D. Pontier, and D. Chessel. 2008. Testing for phylogenetic signal in phenotypic traits: New matrices of phylogenetic proximities. Theoretical Population Biology 73:79–91.

Peñaflor, M. F. G. V., M. Erb, L. A. Miranda, A. G. Werneburg, and J. M. S. Bento. 2011. Herbivore-induced plant volatiles can serve as host location cues for a generalist and a specialist egg parasitoid. Journal of Chemical Ecology 37:1304–1313.

Pérez, J., J. C. Rojas, P. Montoya, P. Liedo, and A. Castillo. 2013. *Anastrepha* egg deposition induces volatiles in fruits that attract the parasitoid *Fopius arisanus*. Bulletin of Entomological Research 103:318–325.

Perez-Hedo, M., P. Urbaneja-Bernat, J. A. Jaques, V. Flors, and A. Urbaneja. 2015. Defensive plant responses induced by *Nesidiocoris tenuis* (Hemiptera: Miridae) on tomato plants. Journal of Pest Science 88:543–554.

Petitt, F., T. Turlings, and S. Wolf. 1992. Adult experience modifies attraction of the leafminer parasitoid *Opius dissitus* (Hymenoptera: Braconidae) to volatile semiochemicals. Journal of Insect Behavior 5:623–634.

Pettersson, E. M. 2001. Volatile attractants for three Pteromalid parasitoids attacking concealed spruce bark beetles. Chemoecology 11:89–95.

Potting, R. P. J., L. E. M. Vet, and M. Dicke. 1995. Host microhabitat location by stem-borer parasitoid *Cotesia flavipes* - the role of herbivore volatiles and locally and systemically induced plant volatiles. Journal of Chemical Ecology 21:525–539.

Raghava, T., P. Ravikumar, R. Hegde, and A. Kush. 2010. Spatial and temporal volatile organic compound response of select tomato cultivars to herbivory and mechanical injury. Plant Science 179:520–526.

Rani, P. U., and K. Sandhyarani. 2012. Specificity of systemically released rice stem volatiles on egg parasitoid, *Trichogramma japonicum* Ashmead behaviour. Journal of Applied Entomology 136:749–760.

Reed, H. C., R. D. K., and N. C. Elliott. 1992. Comparative life table statistics of *Diaeretiella rapae* and *Aphidius matricariae* on the russian wheat aphid. Southwestern Entomologist 17:307–312.

Rogers, M. E., and D. A. Potter. 2003. Effects of spring imidacloprid application for white grub control on parasitism of Japanese beetle (Coleoptera: Scarabaeidae) by *Tiphia vernalis* (Hymenoptera: Tiphiidae). Journal of Economic Entomology 96:1412–1419.

Rousse, P., E. Harris, and S. Quilici. 2005. *Fopius arisanus*, an egg-pupal parasitoid of Tephritidae. Overview. Biocontrol News and Information 26:59–69.

Salerno, G., F. De Santis, A. Iacovone, F. Bin, and E. Conti. 2013. Short-range cues mediate parasitoid searching behavior on maize: the role of oviposition-induced plant synomones. Biological Control 64:247–254.

Samson, P. 1984. The biology of *Roptrocerus xylophagorum* [Hym.: Torymidae], with a note on its taxonomic status. Entomophaga 29:287–298.

Sanders, C. J., J. K. Pell, G. M. Poppy, A. Raybould, M. Garcia-Alonso, and T. H. Schuler. 2007. Host-plant mediated effects of transgenic maize on the insect parasitoid *Campoletis sonorensis* (Hymenoptera: Ichneumonidae). Biological Control 40:362–369.

Schwartz, A., and D. Gerling. 1974. Adult biology of *Telenomus remus* [Hymenoptera: Scelionidae] under laboratory conditions. Entomophaga 19:483–492.

Sengonca, Ç., and G. Peters. 1993. Biology and effectiveness of *Apanteles rubecula* Marsh.(Hym., Braconidae), a solitary larval parasitoid of *Pieris rapae* (L.)(Lep., Pieridae). Journal of Applied Entomology 115:85–89.

Shiojiri, K., J. Takabayashi, S. Yano, A. Takafuji, et al. 2000. Flight response of parasitoids toward plant-herbivore complexes: a comparative study of two parasitoid-herbivore systems on cabbage plants. Applied Entomology and Zoology 35:87–92.

Signoretti, A. G. C., M. F. G. V. Penaflor, L. S. D. Moreira, N. C. Noronha, and J. M. S. Bento. 2012. Diurnal and nocturnal herbivore induction on maize elicit different innate response of the fall armyworm parasitoid, *Campoletis flavicincta*. Journal of Pest Science 85:101–107.

Sime, K. R., K. M. Daane, H. Nadel, C. S. Funk, R. H. Messing, J. W. Andrews Jr, M. W. Johnson, and C. H. Pickett. 2006. *Diachasmimorpha longicaudata* and *D. kraussii* (Hymenoptera: Braconidae), potential parasitoids of the olive fruit fly. Biocontrol Science and Technology 16:169–179.

Souissi, R., J. P. Nenon, and B. Le Ru. 1998. Olfactory responses of parasitoid *Apoanagyrus lopezi* to odor of plants, mealybugs, and plant-mealybug complexes. Journal of Chemical Ecology 24:37–48.

Steidle, J., and M. Schöller. 1997. Olfactory host location and learning in the granary weevil parasitoid *Lariophagus distinguendus* (Hymenoptera: Pteromalidae). Journal of Insect Behavior 10:331–342.

Sullivan, B. T., E. M. Pettersson, K. C. Seltmann, and C. W. Berisford. 2000. Attraction of the bark beetle parasitoid *Roptrocerus xylophagorum* (Hymenoptera: Pteromalidae) to host-associated olfactory cues. Environmental Entomology 29:1138–1151.

Takabayashi, J., S. Takahashi, M. Dicke, and M. A. Posthumus. 1995. Developmental stage of herbivore *Pseudaletia separata* affects production of herbivore-induced synomone by corn plants. journal of Chemical Ecology 21:273–287.

Tamiru, A., T. J. A. Bruce, C. M. Woodcock, J. C. Caulfield, C. A. O. Midega, C. K. P. O. Ogol, P. Mayon, M. A. Birkett, J. A. Pickett, and Z. R. Khan. 2011. Maize landraces recruit egg and larval parasitoids in response to egg deposition by a herbivore. Ecology Letters 14:1075–1083.

Tamo, C., I. Ricard, M. Held, A. C. Davison, and T. C. J. Turlings. 2006. A comparison of naive and conditioned responses of three generalist endoparasitoids of lepidopteran larvae to host-induced plant odours. Animal Biology 56:205–220.

Thompson, W. R. 1951. A Catalogue Of Parasites And Predators Of Insect Pests, Section 2; Host Parasite Catalogue, Part. 1. Hosts Of The Coleoptera and Diptera. Commonwealth Agricultural Bureau; London.

———. 1953. A Catalogue Of Parasites And Predators Of Insect Pests, Section 2; Host Parasite Catalogue, Part. 2. Hosts Of The Hymenoptera (Agaonidae to Braconidae). Commonwealth Agricultural Bureau; London.

———. 1955. A Catalogue Of Parasites And Predators Of Insect Pests, Section 2; Host Parasite Catalogue, Part. 3. Hosts Of The Hymenoptera (Calliceratid To Evanid). Commonwealth Agricultural Bureau; London.

———. 1957. A Catalogue Of Parasites And Predators Of Insect Pests, Section 2; Host Parasite Catalogue, Part. 4. Hosts Of The Hymenoptera (Ichneumonidae). Commonwealth Agricultural Bureau; London.

———. 1958. A Catalogue Of Parasites And Predators Of Insect Pests, Section 2; Host Parasite Catalogue, Part. 5. Hosts Of The Hymenoptera (Miscogasteridae To Trigonalidae). Commonwealth Agricultural Bureau; London.

Tillinger, N. A., G. Hoch, and A. Schopf. 2004. Effects of parasitoid associated factors of the endoparasitoid *Glyptapanteles liparidis* (Hymenoptera: Braconidae). European Journal of Entomology 101:243–250.

Torres, J. B., D. L. Musolin, and J. C. Zanuncio. 2002. Thermal requirements and parasitism capacity of *Trissolcus brochymenae* (Ashmead)(Hymenoptera: Scelionidae) under constant and fluctuating temperatures, and assessment of development in field conditions. Biocontrol Science and Technology 12:583–593.

Turlings, T., P. Mccall, H. Alborn, and J. Tumlinson. 1993. An elicitor in caterpillar oral secretions that induces corn seedlings to emit chemical signals attractive to parasitic wasps. Journal of Chemical Ecology 19:411–425.

Van Poecke, R., M. Posthumus, and M. Dicke. 2001. Herbivore-induced volatile production by *Arabidopsis thaliana* leads to attraction of the parasitoid *Cotesia rubecula*: Chemical, behavioral, and gene-expression analysis. Journal of Chemical Ecology 27:1911–1928.

Virla, E. G. 2001. Notes on the biology of *Anagrus breviphragma* (Hymenoptera, Mymaridae), natural enemy of the corn lefhopper *Dalbulus maidis* (Hemiptera, Cicadellidae) and others plant diseases vectors in South America. Boletín de Sanidad Vegetal. Plagas 27:239–248.

Wharton, R., and P. Marsh. 1978. New world Opiinae (Hymenoptera: Braconidae) parasitic on Tephritidae (Diptera). Journal of the Washington Academy of Sciences 68:147–167.

Wishart, G., and E. Monteith. 1954. *Trybliographa rapae* (Westw.)(Hymenoptera: Cynipidae), a parasite of *Hylemya* spp.(Diptera: Anthomyiidae). The Canadian Entomologist 86:145–154.

Wojcik, B., W. H. Whitcomb, and D. H. Habeck. 1976. Host range testing of *Telenomus remus* (Hymenoptera: Scelionidae). Florida Entomologist pages 195–198.

Wyckhuys, K. A. G., and G. E. Heimpel. 2007. Response of the soybean aphid parasitoid *Binodoxys communis* to olfactory cues from target and non-target host-plant complexes. Entomologia Experimentalis et Applicata 123:149–158.

Wyckhuys, K. A. G., J. E. Strange-George, C. A. Kulhanek, F. L. Wäckers, and G. E. Heimpel. 2008. Sugar feeding by the aphid parasitoid *Binodoxys communis*: How does honeydew compare with other sugar sources? Journal of Insect Physiology 54:481–491.

Yan, Z. G., Y. H. Yan, and C. Z. Wang. 2005. Attractiveness of tobacco volatiles induced by *Helicoverpa armigera* and *Helicoverpa assulta* to *Campoletis chlorideae*. Chinese Science Bulletin 50:1334–1341.

Yean, W., and P. Xiongfei. 1986. Investigation of the host range of *Anagrus nilaparvatae* (Hymenoptera: Mymaridae). Natural Enemies of Insects 4:010.

Yeargan, K. 1982. Reproductive capability and longevity of the parasitic wasps *Tenomus podisi* and *Trissolcus euschisti*. Annals of the Entomological Society of America 75:181–183.

Yu, D. S. K. 2012. Taxapad Ichneumonoidea. http://www.ichneumonoidea.name/.

Yu, H., Y. Zhang, K. A. Wyckhuys, K. Wu, X. Gao, and Y. Guo. 2010. Electrophysiological and behavioral responses of *Microplitis mediator* (Hymenoptera: Braconidae) to caterpillar-induced volatiles from cotton. Environmental Entomology 39:600–609.

Zanuncio, J. C., M. Neto, F. da Costa, W. d. S. Tavares, I. Cruz, G. L. D. Leite, and J. E. Serrão. 2013. Functional and numerical responses and reproduction of *Campoletis flavicincta* parasitizing *Spodoptera frugiperda* caterpillars. Acta Scientiarum. Agronomy 35:419–426.

Zaviezo, T., and N. Mills. 1999. Aspects of the biology of *Hyssopus pallidus* (Hymenoptera: Eulophidae), a parasitoid of the codling moth (Lepidoptera: Olethreutidae). Environmental Entomology 28:748–754.

Zhu, P., G. M. Gurr, Z. Lu, K. Heong, G. Chen, X. Zheng, H. Xu, and Y. Yang. 2013. Laboratory screening supports the selection of sesame (*Sesamum indicum*) to enhance *Anagrus* spp. parasitoids (Hymenoptera: Mymaridae) of rice planthoppers. Biological Control 64:83–89.
